# Establishment of a new method for precisely determining the functions of individual mitochondrial genes, using *Dictyostelium *cells

**DOI:** 10.1186/1471-2156-9-25

**Published:** 2008-03-21

**Authors:** Junji Chida, Aiko Amagai, Masashi Tanaka, Yasuo Maeda

**Affiliations:** 1 Department of Developmental Biology and Neurosciences, Graduate School of Life Sciences, Tohoku University, Aoba, Sendai 980-8578, Japan; 2 Division of Enzyme Chemistry, Institute for Enzyme Research, Tokushima University, Tokushima, Tokushima 770-8503, Japan; 3 Genomics for Longevity and Health, Tokyo Metropolitan Institute of Gerontology, 35-2 Sakae-cho, Itabashi-ku, Tokyo 173-0015, Japan

## Abstract

**Background:**

Disruption of mitochondrial genes may become a powerful tool for elucidating precisely the functions of individual mitochondrial genes. However, it is generally difficult to manipulate genetically mitochondrial genes, because 1) a mitochondrion is surrounded by inner and outer membranes, and 2) there are a large number of mtDNA copies in a single cell. This is the reason why we tried to establish a novel method for disrupting a certain mitochondrial gene (*rps4*), using *Dictyostelium *cells.

**Results:**

Here, we have developed a new method for specifically disrupting a mitochondrial gene (*rps4 *; ribosomal protein subunit S4), by a combination of homologous recombination and delivery of an appropriate restriction endonuclease (*Sfo*I) into mitochondria. First, mitochondrially targeted *Sfo*I whose expression is under control of the tetracycline (Tet)-regulated gene expression system was introduced into cells heteroplasmic with respect to the *rps4 *gene. Then, the heteroplasmic cells were produced by homologous recombination by use of the construct in which the unique *Sfo*I site and the 5'-half of the *rps4 *coding region were deleted not to be digested by *Sfo*I, and therefore their mitochondria have both the wild-type mtDNA and the mutant mtDNA with the disrupted *rps4 *gene. In response to removal of Tet from growth medium, *Sfo*I was selectively delivered into mitochondria and digested only the wild-type mtDNA but not the mutated *rps4*. Thus one can gain *rps4*-null cells with only the mutated mtDNA, under the Tet-minus condition.

**Conclusion:**

The mitochondrial gene-disruption method presented here must be widely useful for precisely determining the functions of individual mitochondrial genes. This is the first report to demonstrate complete and specific mitochondrial gene disruption.

## Background

Mitochondrial DNA (mtDNA) is a maternally inherited circular genome present in hundreds to thousands of copies in most cells. A single mitochondrion contains hundreds of proteins that together are responsible for the various biological functions of the organelle. Most of these proteins are encoded in the nucleus, synthesized in the cytosol, and then directed to the mitochondria by specific targeting sequences (mitochondria targeting sequence; MTS) to be inserted in the mitochondrion by a complex import machinery largely conserved across species. Mitochondrial functions are generally presented as the central pathway for energy metabolism including ATP synthesis through respiration, but several other aspects of mitochondrial function have been noted, such as key roles in apoptosis, free radical production, thermogenesis and calcium signaling. In addition, increasing evidence indicates that mitochondria possess novel and critical functions as the regulatory machinery of the growth/differentiation transition (GDT), cell movement, cell-type choice, and pattern formation, as exemplified well in *Dictyostelium*, a wonderful model organism in the field of cell and developmental biology [1]. For example, the expression of the mitochondrial ribosomal protein (*rps4*) that is encoded by mtDNA is required for differentiation from the GDT point in the cell cycle [2].

The mtDNA of *Dictyostelium discoideum *is a 55.6-kb circular double-stranded DNA molecule which encodes 2 rRNAs, 10 subunits of the NADH dehydrogenase complex (NAD1, 2, 3, 4, 4L, 5, 6, 7, 9, and 11), apocytochorome *b *(cyt *b*), 3 subunits of cytochrome *c *oxidase (Cox 1, 2, and 3), 15 ribosomal proteins, and 5 other open reading frames (ORFs), excluding intronic ORFs (essential peptides of enzymes for oxidative phosphorylation, 3 rRNAs and 18 tRNAs) [3,4]. The mitochondrial gene cluster (*dia3*) including *rps4 *is specifically expressed in response to starvation around the GDT point and plays a critical role in the initiation of differentiation in *D. discoideum *Ax-2 cells. The *rps4 *gene is present as a single copy in mtDNA, but the copy number is multiple because numerous mitochondria are contained in a cell. In spite of this situation, we attempted homologous recombination to determine the function of *rps4*, inactivating the subpopulation of the *rps4 *gene [2]. The homologous recombination was beautifully accomplished with high efficiency and specificity, and resulting G418-resistant transformants exhibited mtDNA heteroplasmy with both the wild-type *rps4 *and its inactivated gene. The partial inactivation of the *rps4 *gene greatly impaired differentiation, including cell aggregation. Provided that it is possible to completely inactivate *rps4 *expression, one can expect that the *rps4*-null cells would never differentiate from the GDT point in response to starvation. This is the principal reason why we tried to establish an efficient system for specifically disrupting a mitochondrial gene such as *rps4 *in the present work.

In the present work, we first aimed to preparing transformants in which a certain portion of the *rps4 *gene was disrupted by homologous recombination. For this purpose, a single *Sfo*I site located in the upstream region of *rps4 *and a 5' half of the *rps4*-coding region were deleted by *Bam*HI and *Sma*I digestion so that the deleted *rps4 *(mutant *rps4*; Mut-mtDNA) gene is no longer able to be digested by *Sfo*I. The vector constructs for homologous recombination were introduced into a transformant in which MTS-*Sfo*I is expressed under control of the tetracycline (Tet)-regulated gene expression system to produce heteroplasmic cells with respect to the *rps4 *gene. After removal of Tet from the growth medium, MTS-*Sfo*I was expressed and imported into the mitochondria where it digested the *Sfo*I site of wild-type mtDNA (Wt-mtDNA) to eliminate Wt-mtDNA. Since the Mut-mtDNA was not digested by *Sfo*I, the ratio of Mut-mtDNA/Wt-mtDNA increased strikingly during the growth of the heteroplasmic cells under the Tet-minus condition. This resulted in the creation of Wt-mtDNA-null cells, that is *rps4*-null cells. The method presented here gives us a powerful tool for targeted mitochondrial-gene disruption and therefore for elucidation of the precise function of each mitochondrial gene.

## Results and Discussion

### Creation of ρ^0 ^cells by delivering *Eco*RI into mitochondria

**Figure 1:**
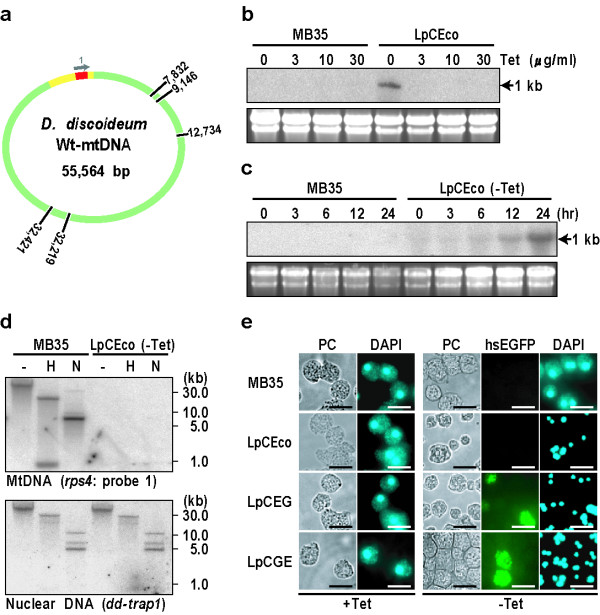
**Elimination of *D. discoideum *mtDNA by introduction of mitochondrion-targeted *Eco*RI**. **(a) **Diagram of Wt-mtDNA indicating the positions of five *Eco*RI sites. The yellow region represents the *dia3 *gene cluster (about 6.5 kb) including the *rps4 *gene (red region). A gray arrow "1" corresponds to the *rps4 *gene-specific oligonucleotide probe used for Southern blot analysis. **(b) **Dose-response analysis of Tet-regulated *Eco*RI mRNA expression. LpCEco and parental MB35 cells were separately grown by shake culture for 24 hours in PS medium containing the designated concentrations of tetracycline (Tet; μg/ml), and the total RNA was extracted for Northern blot analysis using the pCoxIV-*Eco*RI-specific probe. It is evident that the *Eco*RI mRNA (about 1 kb) is expressed in LpCEco cells only under the Tet-minus condition. In the bottom of each lane, the amounts of ribosomal 17S and 26S RNA stained with EtBr are shown as loading control. **(c) **Kinetics of *Eco*RI mRNA induction during 24 hours of incubation without Tet. MB35 and LpCEco cells were grown in Tet-minus PS medium for the indicated times (hr), followed by Northern blotting. The *Eco*RI mRNA begins to be expressed in LpCEco cells 12 hours after removal of Tet and increase to the peak level after 24 hours of incubation. **(d) **Mitochondrially tagged *Eco*RI leads to almost complete degradation of mtDNA. Total DNAs were extracted from MB35 and LpCEco cells 48 hours after removal of Tet and digested with the indicated restriction enzymes (H, *Hin*dIII; N, *Nde*I), followed by electrophoretic size-fractionation and Southern blot analysis using either the ^32^P-labeled nuclear DNA-specific probe (*dd-trap1*) or the mtDNA-specific probe (*rps4*). It is clear that LpCEco cells grown without Tet are almost completely devoid of mtDNA. **(e) **Staining patterns of MB35, LpCEco, LpCEG, and LpCGE cells with DAPI. Cells were cultured in PS medium in the presence (+Tet) or absence (-Tet) of Tet for 48 hours. Under the Tet-plus conditions, the nuclei and mitochondria are stained well with DAPI as large dots and surrounding small granules respectively, in all of the cells examined. Under the Tet-minus conditions, however, the DAPI-staining of mitochondria disappears almost completely from LpCEco, LpCEG and LpCGE cells but not from parental MB35 cells, though the staining of the nucleus is retained well after removal of Tet. Bars, 10 μm.

As the first step to be tested, we needed to know if conditional targeting of restriction endonucleases such as *Eco*RI into *Dictyostelium *mitochondria can eliminate mtDNA completely to give mtDNA-null cells (ρ^0^-cells). In the Wt-mtDNA there are 5 *Eco*RI sites (Figure 1a). To deliver *Eco*RI into mitochondria, the *Eco*RI gene was fused with the presequence that encodes the signal peptide (MTS) of cytochrome *c *oxidase subunit IV (pCoxIV), and then the fusion gene was inserted into a *Dictyostelium *TRE-P_min _(Tet-mediated promoter)-plasmid (pCE38) shown in Additional data file 1b. Subsequently, *Dictyostelium *Tet-Off cells (MB35 cells) were transformed with the pMB38/pCoxIV-*Eco*RI (pCE38) vector, and the resulting double-transformants were clonally selected in the presence of 20 μg/ml of tetracycline (Tet), 10 μg/ml of blasticidin S and 50 μg/ml of G418. In a total of about 600 clones examined, the pCox-*Eco*RI expression after removal of tetracycline was found to vary widely, and most of clones exhibited only poor induction of pCox-*Eco*RI under the tetracycline-minus (-Tet) conditions (data not shown). The inefficient expression in the Tet-Off system is possibly due to unfit integration of the transactivator plasmid (pMB35) [5] and response plasmid (pCE38). A wide range of the expression levels was also reported in essentially the same Tet-Off system using the TRE-P_min_/luciferase [5]. Fortunately, we could obtain one blasticidin S-resistant clone (referred to as LpCEco cells) in which the mRNA of *Eco*RI was expressed at a high level within 24 hours after removal of Tet. We initially needed about 600 clones to generate a doubly-transformed cell line. Recently, however, we have succeeded in obtaining the doubly-transformed cells (LpCEco cells or LpCSfo cells) much more efficiently by the use of MB35 cells that are strictly regulated by the Tet-Off system, as noted in methods.

Dose-response analysis of MTS-*Eco*RI mRNA expression levels was done by use of LpCEco cells and parental MB35 cells, both of which were exposed to various Tet-concentrations ranging from 0 μg/ml to 30 μg/ml during 24 hours of incubation in growth (PS) medium. As shown in Figure 1b, the MTS-*Eco*RI was expressed only in LpCEco cells incubated under the -Tet condition. Figure 1c shows a time course of the MTS-*Eco*RI mRNA expression in LpCEco cells and parental MB35 cells after complete removal of Tet from PS-medium: the mRNA began to be expressed 12 hours after removal of Tet in LpCEco cells and reached maximal levels by 24 hours of incubation. Incidentally, LpCEco cells incubated under the -Tet condition completely lost the ability to grow in PS medium within 48 hours of incubation.

To know if mtDNA in LpCEco cells is actually eliminated 48 hours after removal of Tet, Southern blot analysis was carried out using the nuclear DNA-specific probe (*dd-trap1*) or mtDNA-specific probe (*rps4*). From densitometric measurements of the autoradiograms obtained, the mtDNA in LpCEco cells was found to be almost completely eliminated, but no difference in the amount of the nuclear DNA was detected between LpCEco cells and parental MB35 cells (Figure 1d). This indicates that LpCEco cells are efficiently converted to the ρ^0^-state within 48 hours after removal of Tet. Similar observations have been reported in human 293T and cybrid NARP cells [6,7], indicating that restriction-enzyme mediated destruction of mtDNA is an all-or-none process.

When parental MB35 cells were stained with DAPI, their nuclei and mitochondria were stained as relatively large dots and small granules, irrespective of the presence or absence of Tet (Figure 1e). Essentially the same staining pattern was attained in LpCEco cells (referred to as LpCEco (+Tet) cells) grown for 48 hours in the presence of Tet (+Tet). In LpCEco cells (referred to as LpCEco (-Tet) cells) grown for 48 hours under the -Tet condition, however, DAPI-staining of mitochondria was never observed, though the staining of nuclei was retained well (Figure 1e). This was also the case in LpCEG and LpCGE cells that express fusion proteins consisting of MTS-*Eco*RI and hsEGFP (Additional data file 2). When LpCEG and LpCGE cells were examined under fluorescence microscopy, the fluorescence of hsEGFP was confirmed to locate in the mitochondria of some cells grown without Tet (Figure 1e). Therefore, both of *Eco*RI-hsEGFP and hsEGFP-*Eco*RI fusion proteins were concluded to be properly targeted to mitochondria in a Tet-regulated manner. During incubation of LpCEG and LpCGE cells under the -Tet condition, there were transiently observed a wide range of variation in intramitocondrial hsEGFP expression, and more or half of cells in the field of Figure 1e seemed to have little or no fuorecsence. Thus, although there were a significant number of cells in which the expression of hsEGFP is low, there are five *Eco*RI sites in mtDNA, and also *Eco*RI has very high activity to cleave mtDNA. The cleaved mtDNA has no duplication ability and is eventually digested in mitochondria, thus resulting in generation of ρ^0^-state cells. We detected no signs of apoptosis, such as the formation of DNA ladders (Figure 1d) or the appearance of nuclear fragmentation and condensation (Figure 1e), in the mtDNA-null cells (ρ^0 ^cells). These results indicate that the restriction enzyme imported into mitochondria is tightly retained within the mitochondrial matrix compartment without attacking the nuclear genome.

### Depletion of mtDNA induces mitochondrial dysfunction

**Figure 2:**
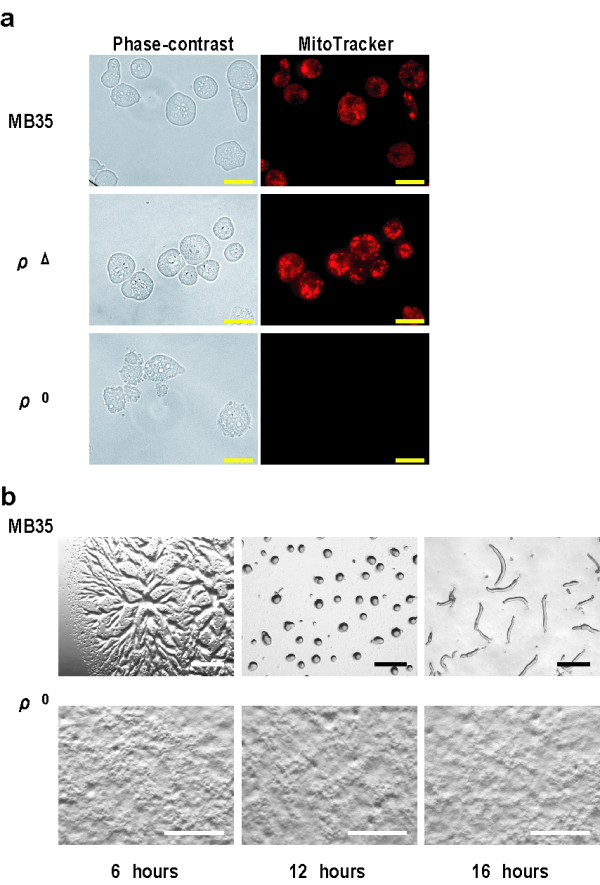
**Phenotypes of ρ^0 ^cells, ρ^Δ ^cells, and parental MB35 cells**. **(a) **Membrane potential of mitochondria. The membrane potential of mitochondria was monitored by staining of cells with the cationic dye MitoTracker Orange CMTMRos (MTK) at a concentration of 0.5 μM for 30 minutes. For comparison, we show here the membrane potential of mitochondria in ρ^Δ ^cells with a reduced amount of mtDNA, which were produced by incubating LpCEco cells in Tet-minus growth medium for a relatively short period (8 hours). Rather stronger staining by MitoTracker Orange is observed in many of mitochondria contained in the ρ^Δ ^cells compared to MB35 cells. In contrast, the mitochondrial membrane potential is completely vanished in ρ^0 ^cells that were produced by incubating LpCEco cells in Tet-minus growth medium for a relatively long period (48 hours). Bars, 20 μm. **(b) **Development of starved MB35 cells and ρ^0 ^cells on agar. MB35 cells and ρ^0 ^cells were washed twice in BSS and plated on 1.5% non-nutrient agar at a density of 5 × 10^6 ^cells/cm^2^. This was followed by incubation for the indicated times at 22°C. Bars, 0.5 mm.

We have previously reported that ρ^Δ ^cells with a reduced amount (25%) of mtDNA, which were produced from parental Ax-2 cells by exposing them to ethidium bromide (EtBr), exhibit a series of developmental defects, such as delay of differentiation and abnormal cell patterning after starvation [8]. Therefore the developmental phenotypes of ρ^0 ^cells (LpCEco cells grown for 48 hours under the -Tet condition) were examined and compared with those of parental MB35 cells and ρ^Δ ^cells that were prepared by a relatively short period (8 hours) of growth under the -Tet condition. With respect to the membrane potential of mitochondria, mitochondrial staining with MitoTracker Orange was found to be rather stronger in the ρ^Δ ^cells than in MB35 cells (Figure 2a), as observed in ρ^Δ ^cells produced by EtBr-exposure [8]. By contrast, the membrane potential-dependent staining with MitoTracker Orange was completely abolished in ρ^0 ^cells (Figure 2a). Electronmicroscopic observations showed that mitochondria in ρ^Δ ^cells exert marked structural transformation, and that ρ^0 ^cells contain quite abnormal mitochondria in which cristae are highly disorganized (data not shown). In this connection, progressive external opthalmoplegia and Kearns-Sayre syndrome, which are caused by deletion of mtDNA [9,10], are known to exert marked morphological changes in the mitochondria of muscle tissues [11]. When the ρ^0 ^cells and parental MB35 cells were starved and incubated on 1.5% non-nutrient agar, the former showed no sign of cell aggregation even after 60 hours of incubation at 22°C (Figure 2b). Therefore, it is most likely that at least 25% of the mtDNA might be required for maintenance of cell growth. The ρ^Δ ^cells also exhibit a series of fascinating behaviors after starvation: they show a marked delay of differentiation including cell aggregation and abnormal cell-type proportioning [8], thus suggesting strongly the importance of mtDNA in a variety of cellular functions. One conclusion to be drawn is that neither growth nor differentiation occurred in the ρ^0 ^cells, presumably because of ATP depletion. Interestingly, the mitochondrial membrane potential does not necessarily decrease linearly coupled with reduced levels of mtDNA, and is abruptly eliminated below a certain level of mtDNA (Figure 2a). This phenomenon is called the threshold effect [12]. Taken together the data presented here strongly suggest that the threshold value is around 20–25% in *Dictyosyelium *mitochondria, and that the mtDNA level less than 20% may cause growth arrest and also a failure of cells to differentiate after starvation.

### Strategy for creating *rps4*-null cells by a combination of homologous recombination and targeted *Sfo*I introduction

**Figure 3:**
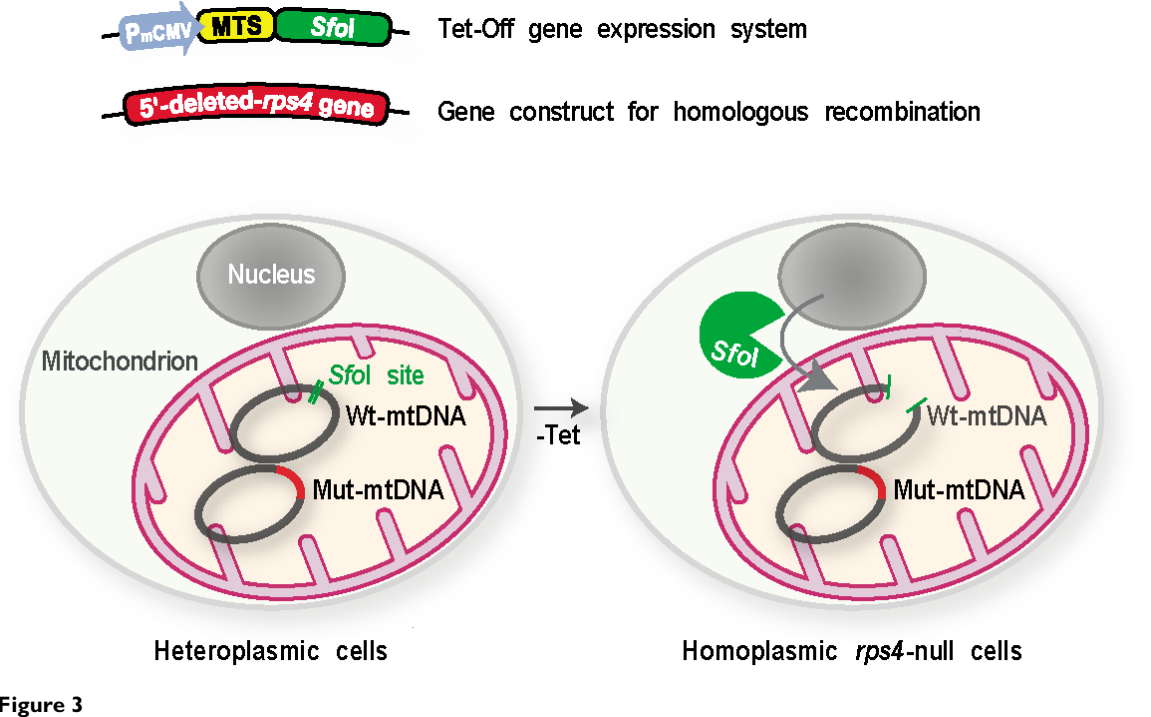
**Strategy for creating *rps4*-null cells**. As a starting material, LpCSfo cells in which pCoxIV (MTS)-*Sfo*I is expressed under the tetracycline-minus (-Tet) condition, were prepared. Subsequently, the mutant *rps4 *gene (Mut-mtDNA for homologous recombination), in which the upstream *Sfo*I-site and a 5'-half of *rps4 *coding region were deleted, was introduced into LpCSfo cells to obtain heteroplasmic transformants (LpCSfo^HR ^cells) with mitochondria consisting of the Mut-mtDNA and wild-type mtDNA (Wt-mtDNA). Coupled with removal of Tet from growth medium, the fusion protein MTS-*Sfo*I synthesized in the cytoplasm is exclusively transferred into mitochondria of LpCSfo^HR ^cells and selectively digests Wt-mtDNA but not Mut-mtDNA. Since the digested Wt-mtDNA is not duplicated, the Mut-mtDNA becomes dominant during the course of growth under the -Tet condition, thus eventually giving *rps4*-null cells.

Impairment of mitochondrial function has been associated with a wide range of severe human disorders, which have been regrouped under the name of mitochondrial diseases [13]. Frequently, mtDNA with pathogenic mutations coexist with Wt-mtDNA (mtDNA heteroplasmy). MtDNA mutations manifest their phenotypes only when the proportion of mutated mtDNAs is high (threshold effect) [12]. To reduce this proportion, an effective therapy for these disorders has been tried by use of mitochondrially targeted restriction endonucleases to eliminate specifically mutated mtDNAs [6,7,14,15]. The new method presented in this work was devised around the inverse concept. In Wt-mtDNA, there is a single *Sfo*I site in the upstream of *rps4 *gene. This raised the possibility that the conditioned expression of *Sfo*I in mitochondria of the *rps4*-heteroplasmic cells might allow us to create *rps4*-null cells, as schematically shown in Figure 3. To test this possibility, we first needed to prepare a transformant in which    the fusion protein MTS-SfoI   is expressed in response to removal of tetracycline from the PS medium, as in the case of LpCEco cells described in the preceding section. For this purpose, MB35 cells were transformed with the pMB38/pCoxIV-*Sfo*I (pCS38) vector (Additional data file 1c), and the resulting double-transformants were clonally selected in the presence of 20 μg/ml of tetracycline (Tet), 10 μg/ml of blasticidin S and 50 μg/ml of G418. In a total of about 800 clones examined, 24 likely clones were initially obtained as ones that expressed the MTS-*Sfo*I after removal of Tet from growth medium, but most of them were found to exhibit a poor response to Tet. Fortunately, however, we could gain one nice transformant (referred to as LpCSfo cells) that stably expressed the MTS-*Sfo*I coupled with removal of Tet. The method adopted here is based on introduction of *Sfo*I into mitochondria and its exact expression under the -Tet condition. Therefore, the use of MB35 cells in which the Tet-Off system is strictly regulated is quite critical for efficient selection of transformants such as LpCSfo cells.

**Figure 4:**
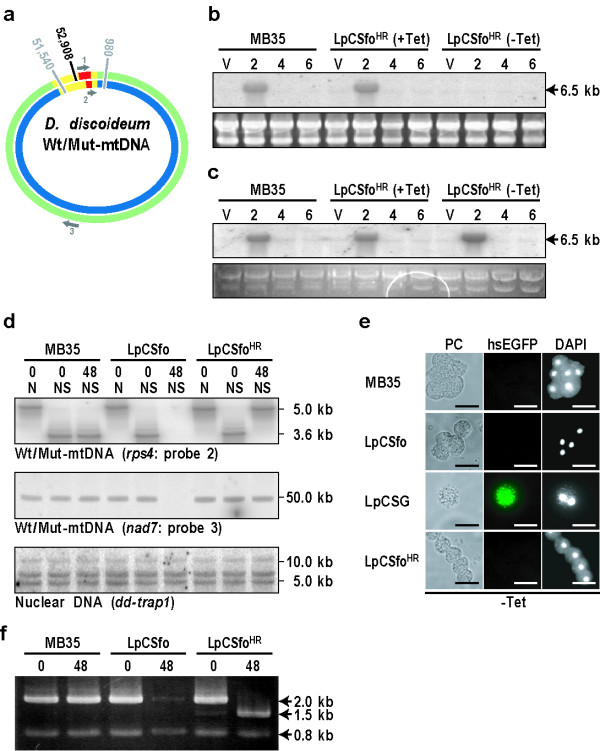
**Rapid and complete shift of LpCSfo^HR ^cells (mtDNA heteroplasmic cells) to a *rps4*-null state by the expression of mitochondrion-targeted *Sfo*I**. **(a) **Diagram of wild-type-mtDNA (Wt-mtDNA; outer green circle) and mutant-mtDNA (Mut-mtDNA; inner blue circle) indicating the positions of the restriction sites (*Sfo*I, 52,908; *Nde*I, 980 and 51,540) and the oligonucleotide probes (gray arrows "1", "2", and "3") used. The *dia3 *gene cluster (yellow line) containing mutated *rps4 *gene (red line) in Mut-mtDNA is slightly shorter than that in Wt-mtDNA, because the *Sfo*I site and a 5'-half of *rps4 *coding region were deleted. **(b) **MB35 cells and LpCSfo^HR ^cells grown with (+Tet) or without (-Tet) tetracycline were washed twice in BSS and shaken in BSS for the indicated times (hr) at 22°C. This was followed by extraction of the total RNA and Northern hybridization using the probe "1" that covers the whole *rps4 *gene. The expression of *dia3 *gene in MB35 cells is usually transient and reaches to the maximum level after about 2 hours of starvation in BSS. The large polycistronic *dia3 *transcript (6.5 kb) was detected in LpCSfo^HR ^(+Tet) cells, because they have heteroplasmically Wt-mtDNA and Mut-DNA under the +Tet condition. In LpCSfo^HR ^cells grown under the -Tet condition, however, only Wt-mtDNA but not Mut-mDNA was selectively cleaved by *Sfo*I, thus resulting in formation of *rps4*-null cells. Actually, Wt-mtDNA (wild-type *dia3 *transcript) as detected by the probe "1" was never observed in LpCSfo^HR ^(-Tet) cells. V, vegetative growth phase. **(c) **When the *dia3 *transcripts from Wt-mtDNA and/or Mut-mtDNA were hybridized by the probe "2", they gave a positive band at the position of about 6.5 kb, because this probe must detect the mutated *dia3 *transcript with a slightly shorter length as well as the wild-type *dia3 *transcript. **(d) **Southern blot analysis of total DNAs extracted from MB35, LpCSfo and LpCSfo^HR ^cells. DNAs were digested with the indicated restriction enzymes (N, *Nde*I; NS, *Nde*I and *Sfo*I), followed by electrophoresis and Southern blot analysis, as described in the legend of Figure 1d. The numbers shown below the cells used indicate incubation times (hr) in PS medium under the Tet-minus conditions. As probes, the probe "2" (for mitochondrial *rps4*), "3" (mitochondrial *nad7 *as a region other than *rps4*) and a specific probe for nuclear *dd-trap1 *were used. Since LpCSfo cells grown without tetracycline lose their mtDNA and become ρ^0 ^cells, the whole mtDNA (about 50 kb) detected by the probe "3" disappeared. Accordingly, bands of 5 kb and 3.6 kb as detected by the probe "1" were never observed in the ρ^0 ^cells. As was expected, almost the same amount of the nuclear gene *dd-trap1 *was detected in all of the samples examined. **(e) **Staining of MB35, LpCSfo, LpCSG and LpCSfo^HR ^cells with DAPI. Cells were grown in PS medium in the absence of Tet for 48 hours at 22°C and stained with DAPI. LpCSfo cells are completely devoid of mitochondrial DAPI-staining, though dot-like stains of nuclei are retained under the -Tet condition. In LpCSG cells, the hsEGFP signal with *Sfo*I was specifically observed in mitochondria. Mitochondria in LpCSfo^HR ^cells were stained with DAPI, because of the presence of Mut-mDNA. Bars, 10 μm. **(f) **PCR analysis of Wt-mtDNA and Mut-mtDNA in MB35, LpCSfo and LpCSfo^HR ^cells. Quantitative mitochondrial genomic-PCR analysis was carried out using DA3RP1 and DA3RP2 primers (Additional data file 3). The numbers shown below the cells used indicate incubation times (hr) in PS medium under the Tet-minus conditions. It is evident that the bands of 2 kb (Wt-mtDNA) are shifted to 1.5 kb (Mut-mtDNA) under the Tet-minus conditions in LpCSfo^HR ^cells. To control for equal loading, a 0.8 kb fragment of the *dd-trap1 *gene was amplified at the same time.

Heteroplasmic LpCSfo^HR ^cells, in which both of Wt-mtDNA and Mut-mtDNA lacking the *Sfo*I site and a 5'-half of *rps4 *coding region are intermingled, were prepared by means of homologous recombination using the vector construct as described in Additional data file 3. When LpCSfo^HR ^cells (+Tet) grown with Tet and parental MB35 cells were starved, the large polycistronic *dia3 *transcripts (about 6.5 kb) including the *rps4 *or its deleted gene were transiently expressed after 2 hours of starvation (Figure 4b). In LpCSfo^HR ^(-Tet) cells grown for 48 hours in the absence of Tet, however, the *dia3 *transcript containing the intact *rps4 *gene was never expressed after starvation (Figure 4b), though the smaller *dia3 *transcript with the 5'-deleted *rps4 *gene was expressed 2 hours after starvation (Figure 4c). It is quite difficult to detect the size-difference between the two-types of *dia3 *transcripts, because the deleted region of *rps4 *gene is not so large compared to the whole *dia3 *gene cluster. As shown in Figure 4d, a 5 kb fragment that should be obtained by digestion of the mutant *dia3 *gene cluster with *Nde*I and *Sfo*I was scarcely observed in LpCSfo^HR ^(+Tet) cells grown for 2 weeks in the presence of Tet. This seemed to indicate that a majority of Mut-mtDNA was lost during a prolonged time of growth under the Tet-plus condition. This is consistent with our previous findings [2] that the heteroplasmic state is not stable, and therefore that Wt-mtDNA becomes dominant gradually during culture, possibly because of preferential duplication of Wt-mtDNA compared to Mut-mtDNA. Importantly, however, a small proportion of Mut-mtDNA was found to be enough to create *rps4*-null cells containing only the Mut-mtDNA, because LpCSfo^HR ^(+Tet) cells were converted to LpCSfo^HR ^(-Tet) cells within 48 hours of growth under the Tet-minus condition (Figure 4d). When MB35, LpCSfo and LpCSfo^HR ^cells grown for 48 hours under the -Tet conditions were stained with DAPI, LpCSfo cells were confirmed to be completely devoid of mitochondrial staining, though dot-like stains of nuclei were retained well (Figure 4e). By contrast, since MB35 cells and LpCSfo^HR ^(-Tet) cells have Wt-mtDNA and Mut-mtDNA respectively, their mitochondria were stained well with DAPI (Figure 4e). In LpCSG cells produced by fusing the hsEGFP sequence with MTS-*Sfo*I, the fluorescence was specifically observed in mitochondria. Unexpectedly, however, mitochondrial DAPI-staining was still retained in the LpCSG cells (Figure 4e). This seemed to indicate that the enzyme activity of the relatively small *Sfo*I molecule might be reduced or abolished when fused with hsEGFP, probably because of a steric hindrance. The lack of of wild-type *rps4 *gene in LpCSfo^HR ^cells incubated for 48 hrs under the -Tet condition was confirmed by PCR analysis (Figure 4f). Again, transformed cells with Mut-mtDNA in which only the *rps4 *gene had been mutated were able to grow even in the presence of introduced *Sfo*I. Conversely, wild-type mtDNA (WT-mtDNA) was selectively digested by *Sfo*I, and the ratio of Mut-mtDNA/WT-mtDNA in the heteroplasmic cells was gradually increased. Thus, cells with little or no Mut-mtDNA became a ρ^0^-state and eventually died because of ATP depletion.

### Developmental phenotypes of *rps4*-null cells

**Figure 5:**
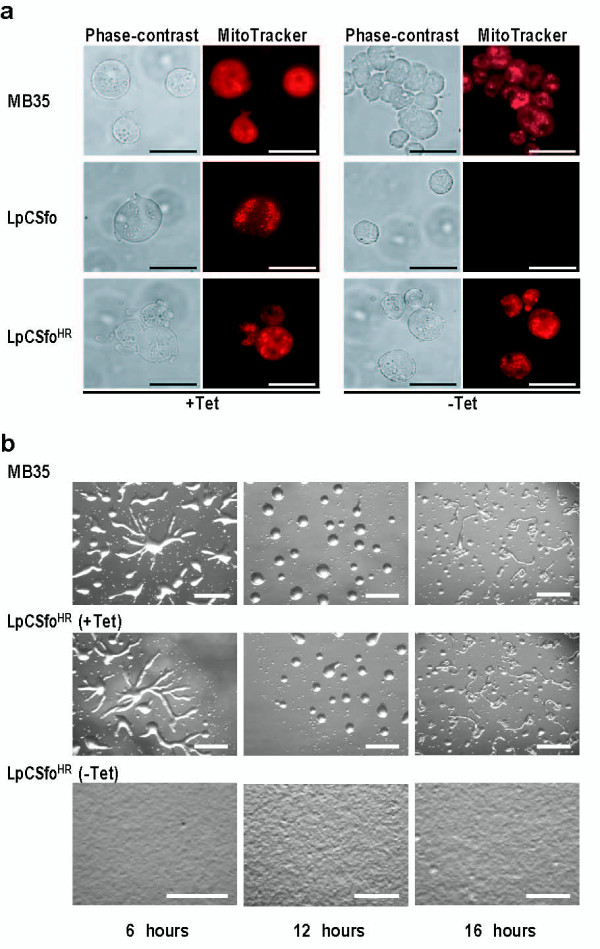
**Phenotypes of MB35 cells, LpCSfo cells, and LpCSfo^HR ^cells**. **(a) **These cells were grown in PS medium with (+Tet) or without (-Tet) teteracyclin. Membrane potential of mitochondria was visualized by staining of cells with MitoTracker Orange, as described in the legend of Figure 2. As was expected, the staining of mitochondria was almost completely vanished in LpCSfo cells grown without Tet, because their mtDNA with an intact *Sfo*I site would be cleaved by *Sfo*I eventually to become a ρ^0 ^state. Bars, 20 μm. **(b) **Development of starved MB35 cells and LpCSfo^HR ^cells on agar. MB35 cells and LpCSfo^HR ^cells grown with (+Tet) or without (-Tet) tetracycline were washed twice in BSS and plated on 1.5% non-nutrient agar at a density of 5 × 10^6 ^cells/cm^2^. This was followed by incubation for the indicated times at 22°C. Bars, 0.5 mm.

LpCSfo cells stopped growing in PS medium within 48 hours after their transfer to Tet-minus medium, because they were converted to a ρ^0 ^state. However, LpCSfo^HR ^(-Tet) cells (*rps4*-null cells) were able to grow normally with almost the same doubling-time as parental MB35 cells, indicating that the intact *rps4 *gene is not required for growth (data not shown). When MB35 cells and LpCSfo^HR ^cells (+Tet) cells were starved and incubated on 1.5% non-nutrient agar at 22°C, they formed aggregation streams after 6 hours and mounds after 12 hours (Figure 5b). This was followed by formation of migrating slugs after 16 hours of incubation. By contrast, starving *rps4*-null cells exhibited a great delay of differentiation: no sign of cell aggregation was noticed even after 16 hours of incubation (Figure 5b), and aggregation-streams were formed only after 60 hours of incubation (data not shown).

When the membrane potential of mitochondria was monitored by staining of cells with MitoTracker Orange, the staining of mitochondria was almost completely abolished in LpCSfo cells grown without Tet, as the case of LpCEco (-Tet) cells (Figure 5a). Here it is of importance to note that mitochondria of LpCSfo^HR ^(-Tet) cells (*rps4*-null cells) were stained well with the dye (Figure 5a), suggesting that the expression of *rps4 *is not necessarily required for the occurrence of mitochondrial membrane potential.

## Conclusion

To our knowledge, this is the first report to demonstrate complete and specific disruption of a certain mitochondrial gene by a combination of homologous recombination and introduction of mitochondrially targeted endonuclease. In the present study, we constructed vectors containing the sequence for *Eco*RI or *Sfo*I downstream of and fused in frame to the mitochondrial targeting sequence (MTS) under control of the Tet-response element and introduced them into *Dictyostelium *cells. This system permits the reversible expression of individual mitochondrial genes in a Tet-regulated manner, to create ρ^0 ^cells or *rps4 *null cells. The usefulness of mitochondrially targeted restriction endonucleases in modulating mtDNA is limited by the presence of an appropriate restriction site. However, this approach may modulate mtDNA heteroplasmy in the context of multiple cleavage sites if expression of the restriction endonuclease could be tightly regulated. In conclusion, we believe that the new method presented here can provide a powerful tool for precisely determining the functions of individual mitiochondrial genes as well as for genetic therapy of mitochondrial diseases.

## Methods

### Culture conditions and transformation of cells

Vegetative cells of *Dictyostelium discoideum *Ax-2 (clone 8A) were axenically grown in growth (PS)-medium, as previously described [8]. The PCR product containing both the mitochondrial targeting sequence (MTS) and endonuclease gene (*Sfo*I or *Eco*RI) was inserted into pMB38 bearing the blasticidin S resistant cassete, which is the response plasmid of tetracycline-regulated gene expression system (Tet-Off system) for *Dictyostelium *cells [5] (Additional data file 1b, 1c, Additional data file 3 and Additional data file 4). The transactivator plasmid of Tet-Off system (MB35) was initially introduced into Ax-2 cells, and the transformed cells (MB35 cells) were selected and cloned with 30 μg/ml G418. The pCE38 or pCS38 vector construct, both of which have TRE- P_min _(tetracycline response element), MTS, endonuclease (*Eco*RI or *Sfo*I) and balasticidin S cassette, was introduced into MB35 cells to obtain LpCEco cells or LpCSfo cells. The transformed cells thus obtained were maintained in PS medium containing 20 μg/ml of tetracycline (Tet), 10 μg/ml of blasticidin S and 50 μg/ml of G418. Recently, we have succeeded in obtaining the doubly-transformed cells (LpCEco cells or LpCSfo cells) much more efficiently by the use of MB35 cells that are strictly regulated by the Tet-Off system. That is, the transformed cells were cloned in 96-well tissue culture plates (Greiner bio-one, Inc., Maybachstr., Frickenhausen, Germany) and incubated in the presence of tetracyclin (+Tet) or in the absence of tetracyclin (-Tet). Subsequently, 20–30 clones that had been able to grow only under the +Tet-condition were isolated, followed by Northern blottings to know if they are LpCEco cells or LpCSfo cells that express the restriction enzyme (*Eco*RI or *Sfo*I).

### Isolation of genomic DNAs and Southern blot analysis

Genomic DNAs were extracted according to the method described previously [16]. Genomic Southern hybridization was performed using the Southern method with a slight modification.

### Isolation of total RNAs and Northern hybridization

Total RNAs were prepared from vegetatively growing cells or cells starved for various periods in 1.5% (wt/vol) non-nutrient agar, using TRIzol Reagent (Life Technologies, Inc., Gaithersburg, MD, USA) as described by the manufacturer. Northern hybridization was performed as described previously [8].

### Staining of cells with a mitochondrion-selective dye, MitoTracker Orange

The vegetatively growing cells were incubated in PS medium containing 0.5 μM MitoTracker Orange CMTMRos (Molecular Probes, Eugene, OR, USA) for 30 minutes at room temperature. After staining with the dye, stained cells were washed twice with BSS. One drop of the cell suspension was put on a cleaned coverslip and cells were fixed with 3.7% (vol/vol) formaldehyde in 20 mM Na/K-phosphate buffer (pH 7.2) for 20 minutes. Subsequently, the cells were washed with PBS (10 mM Na/K-phosphate buffer (pH 7.0), 0.9% (wt/vol) NaCl), as previously described [8]. The stained cells were observed under a fluorescence microscope.

## List of abbreviations

GDT: growth/differentiation transition, mtDNA: mitochondrial DNA, MTS: mitochondrial targeting signal, HR: homologous recombination, *dia3*: differentiation-associated gene 3, *rps4*: ribosomal protein subunit S4 gene, *mtlrRNA*: mitochondrial large ribosomal RNA, TRAP1: tumor necrosis factor receptor-associated protein, NARP: neuronal muscle weakness, ataxia, and retinitis pigmentosa, Tet: tetracycline, EtBr: ethidium bromide

## Authors' contributions

JC performed this research preparing various types of vector constructs. AA and MT initiated and supervised this study. YM carried out electron microscopic studies and wrote the paper. All authors read and approved the final manuscript.

## Supplementary Material

Additional file 1 Schematic maps of the vector constructs used in this work. Click here for file

Additional file 2 *Dictyostelium discoideum *strains used in this study. Click here for file

Additional file 3 Primers used in this study. Click here for file

Additional file 4 Supplementary methods. Click here for file
