# Supplementary material for: Establishment of a new method for precisely determining the functions of individual mitochondrial genes, using Dictyostelium cells
Source: BMC Genet. 2008 Mar 21;9:25. doi: 10.1186/1471-2156-9-25 (PMC2330148; doi:10.1186/1471-2156-9-25)
Supplement: Additional file 1 — Schematic maps of the vector constructs used in this work. [file 1471-2156-9-25-S1.rtf]

Additional data file 1. Schematic maps of the vector constructs used in this work. (a) Map of the integrating transactivator plasmid pMB35 [5]. The plasmid contains sequences for propagation (ColEI ori), ampicillin resistance (ampR) and neomycin (G418) resistance, and a chimeric tetracycline-controled transcriptional activator protein (tTAs) coding gene under control of the D. discoideum actin 15 promoter (A15P), followed by the D. discoideum 2H3T terminator (2H3T). (b, c) Maps of response plasmids pCE38 and pCS38. The plasmid contains sequences for propagation (ColEI ori), a blasticidin S resistance cassette (bsr) under control of the complete D. discoideum actin 15 promoter (A15P), and genes for extrachromosomal maintanance (G4/D5, G5/D6) in D. discoideum (G4/D5, G5/D6). Base 8,086-8,391 correspond to the Tet-responsive element (TRE-Pmin) containing seven copies of the tetO operator sequence of MB38. The gene of EcoRI or SfoI was inserted into the downstream of the cytochrome c oxidase subunit IV (pCoxIV) mitochondrial targeting sequence (MTS). (d, e) To disrupt the mitochondrial rps4 gene by homologous recombination, the vector construct (pBCÄS4) was prepared as follows. pBC14 in which the BamHI-SmaI fragment of D. discoideum mtDNA was inserted into the MCS site of pBluescript SK(+) was digested with SfoI and BstBI to obtain two fragments: one is the region 51,141-55,445 including the 5'-half of rps4 coding region and immediate upstream that contains the SfoI site and trnF (tRNA-coding region), and another is the residual pBC14 . The trnF gene (208 bp) was amplified by PCR from the above SfoI-BstBI fragment, using FT-SmaI and RT-BstBI primers (Additional data file 2) and purified. The PCR product was then digested with SmaI and BstBI, and the fragment was inserted into the above residual pBC14 to obtain pBCÄS4. In the plasmid thus obtained, the SfoI site and the 5'-half of rps4 coding region were deleted, but the trnF gene was retained. (e) The pBCÄS4 was digested by NdeI and SphI, and the linearized NdeI-SphI fragment was introduced into LpCSfo cells by electroporation, to obtain LpCSfoHR cells. nad5, NADH dehydrogenase subunit 5; trnF, tRNA for phenylalanine; rps4, ribosomal protein subunit S4; rps2, ribosomal protein subunit S2. Expected sites of homologous recombination are shown by dotted lines, and the SfoI recognition site as double-green lines.
