# Supplementary material for: Establishment of a new method for precisely determining the functions of individual mitochondrial genes, using Dictyostelium cells
Source: BMC Genet. 2008 Mar 21;9:25. doi: 10.1186/1471-2156-9-25 (PMC2330148; doi:10.1186/1471-2156-9-25)
Supplement: Additional file 2 — Dictyostelium discoideum strains used in this study. [file 1471-2156-9-25-S2.rtf]

Additional data file 2. Dictyostelium discoideum strains used in this study
Strain	Description	Host cell	Reference	
Ax-2
ρΔ
MB35
LpCEco
LpCEG
LpCGE
LpCSfo
LpCSG
LpCSfoHR (+Tet)

LpCSfoHR (-Tet)	Wild-type strain
mtDNA-less strain
pMB35; A15P / tTAs (Neo)
pCE38; TRE-Pmin / pCOXIV-EcoRI (Bsr)
pCEG38;TRE-Pmin / pCOXIV-EcoRI-hEGFP (Bsr)
pCGE38; TRE-Pmin / pCOXIV-hEGFP-EcoRI (Bsr)
pCS38; TRE-Pmin / pCOXIV-SfoI (Bsr)
pCSG38; TRE-Pmin / pCOXIV-SfoI-hEGFP (Bsr)
Heteroplasmic cells containing rps4-inactivated gene in mtDNA
Homoplasmic cells containing rps4-inactivated gene in mtDNA	-
Ax-2
Ax-2
MB35
MB35
MB35
MB35
MB35
LpCSfo

LpCSfo	-
[8]
[5]
This study
This study
This study
This study
This study
This study

This study	
