# Supplementary material for: Establishment of a new method for precisely determining the functions of individual mitochondrial genes, using Dictyostelium cells
Source: BMC Genet. 2008 Mar 21;9:25. doi: 10.1186/1471-2156-9-25 (PMC2330148; doi:10.1186/1471-2156-9-25)
Supplement: Additional file 3 — Primers used in this study. [file 1471-2156-9-25-S3.rtf]

Additional data file 3. Primers used in this study
Primer	Oligonucleotide	Reference	
XhoI-EcoRI-U
EcoRI-U
EcoRI-ApaI-BamHI-L
ApaI-hEGFP-U
XhoI-hEGFP-U
hEGFP-U
hEGFP-ApaI-L
hEGFP-XhoI-L
DA3RP1
DA3RP2
TRAP1-F
TRAP1-R
M1-F
M1-R
FP-MluI
RP-SphI
FR-BamHI
RR-XhoI
FR-MluI
FS-SmaI
FS-MluI
FT-SmaI
RT-BstBI	5'-CTCGAGCATCTAATAAAAAACAGTCAAATAGGC-3'
5'-GCTCTCTGAGAGCCACTTTCC-3'
5'-GGATCCATTGGGCCCGCTTAGATGTAAGCTGTTCA-3'
5'-GGGCCCGGGTGAGCAAGGGCGAGGAGCTGTTC-3'
5'-CTCGAGGGGTGAGCAAGGGCGAGGAGCTGTTC-3'
5'-CCAGCAGAACACCCCCATCGG-3'
5'-GGGCCCTTACTTGTACAGCTCGTCCATGCCGAG-3'
5'-CTCGAGCCTTGTACAGCTCGTCCATGCCGAGAGTG-3'
5'-CTTACAAAACCACTTCGTAATAA-3'
5'-CTTACAAAACCACTTCGTAATAA-3'
5'-CTCTTAGTAAAGTTATATTGAATAGTGG-3'
5'-CTTACTATATTCATAGGAACTTGGTTTC-3'
5'-CGAAATCGATAAGCTTGATATCGAATTCCTGC-3'
5'-CACAGGAAACAGCTATGACCATGATTACG-3'
5'-TTAACGCGTATGCCTGCAGGTCGACT-3'
5'-GTCCGCATGCTTTCACTACGTCAATCT-3'
5'-GATTACGGATCCGTACTAGCCTACCAATCTATTGAA-3' *
5'-TCCGCTCGAGCATCTATGAATAATACATTGGATGAG-3' *
5'-TTAACGCGTCTAGCCTACCAATCTATTG-3' *
5'-TAGAACCCGGGAGCCTACCAATCTATTGAACT-3' *
5'-TAAACGCGTTTACTTGTACAGCTCGTC-3'
5'-TACCCCGGGGAAGCAAATTTATGCGTTTCC-3'
5'-AATGCATTCGAACTAAATATACACAGCTCACTCC-3'	[6]
[6]
[6]
[6]
[6]
[6]
[6]
[6]
[2]
This study
This study
This study
This study
This study
This study
This study
This study
This study
This study
This study
This study
This study
This study	

* The SfoI coding-sequence patents is owned by New England BioLabs
