# Supplementary material for: Establishment of a new method for precisely determining the functions of individual mitochondrial genes, using Dictyostelium cells
Source: BMC Genet. 2008 Mar 21;9:25. doi: 10.1186/1471-2156-9-25 (PMC2330148; doi:10.1186/1471-2156-9-25)
Supplement: Additional file 4 — Supplementary methods. [file 1471-2156-9-25-S4.rtf]

Additional data file 4. Supplementary methods
Construction of the response plasmids
The fusion gene encoding the presequence of cytochrome c oxidase subunit IV (pCoxIV) from S. cerevisiae and the EcoRI was amplified from pUC19-pCoxIV-EcoRI, by use of the FP-MluI and RP-SphI primer to obtain the pCE38 vector construct. To produce cells expressing endonuclease-GFP fusion proteins, pCoxIV-EcoRI-hsEGFP and pCoxIV-hsEGFP-EcoRI combined fusion genes were amplified by PCR from pUC19-pCoxIV-EcoRI-hsEGFP and pUC19-pCoxIV-hsEGFP-EcoRI respectively by use of the FP-MluI and RP-SphI primer. Subsequently, the fusion genes were inserted into the SphI- and MluI-site of the response plasmid (pMB38) to obtain pCEG38 and pCGE38. Schematic maps of the constructs are shown in Additional data file 1. The plasmids (pCE38, pCEG38, and pCGE38) were digested by EcoRV and NotI and then introduced into MB35 cells by electroporation. The SfoI gene was amplified from the plasmid p19SfoIRM (R0606), which was kindly provided by Dr. Donald G. Comb of the New England Biolabs, by use of the RR-XhoI/FR-BamHI and RR-XhoI/FS-SmaI primer. The PCR products thus obtained were cloned into pUC19-pCoxIV-EcoR I and pUC19-pCoxIV-EcoRI-hsEGFP vector after digestion with XhoI/BamHI (pCS19) and XhoI/SmaI (pCSG19), respectively. The pCoxIV-SfoI and pCoxIV-SfoI-hsEGFP combined fusion genes were amplified by PCR from pCS19 and pCSG19 by use of the FP-MluI/RP-SphI primer and FS-MluI/RP-SphI preimer, respectively. Subsequently, the fusion genes were inserted into the SphI- and MluI-site of the response plasmid (pMB38). Schematic maps of the constructs are shown in Additional data file 1. The resulting plasmids (pCS38 and pCSG38) were digested by EcoRV and NotI and then introduced into MB35 cells by electroporation.

Gene construct for homologous recombination
Dictyostelium mtDNA has a single SfoI site in the upstream region of the rps4 gene. Thereupon, pBC14 in which the BamHI-SmaI fragment of D. discoideum mtDNA had been inserted into the MCS of pBluescript SK(+) was digested with SfoI and BstBI to obtain two fragments: one is the region 51,141-55,445 including the 5'-half of rps4 coding region and immediate upstream that contains the SfoI site and trnF (tRNA-coding region), and another is the residual pBC14. The trnF gene (208 bp) was amplified by PCR from the above SfoI-BstBI fragment, using FT-SmaI and RT-BstBI primers and purified. The PCR product was then digested with SmaI and BstBI, and the fragment was inserted into the above residual pBC14 to obtain pBCDS4. In the plasmid thus obtained, the SfoI site and the 5'-half of rps4 coding region were deleted, but the trnF gene was retained (Additional data file 1d). The pBCDS4 was digested by NdeI and SphI, and the linearized NdeI-SphI fragment was introduced into LpCSfo cells by electroporation, to obtain LpCSfoHR cells.
